# Cross‐Sectional Associations of Integrated Lifestyle‐Related Factors With Intrinsic Capacity in Community‐Dwelling Older Adults: The Kashiwa Cohort Study

**DOI:** 10.1111/ggi.70671

**Published:** 2026-07-15

**Authors:** Weida Lyu, Tomoki Tanaka, Katsuya Iijima

**Affiliations:** ^1^ Institute of Gerontology The University of Tokyo Tokyo Japan; ^2^ Institute for Future Initiatives The University of Tokyo Tokyo Japan

**Keywords:** community‐dwelling older adults, intrinsic capacity, nutrition‐related factors, physical factors, social factors

## Abstract

**Aim:**

Identifying the modifiable lifestyle‐related factors associated with intrinsic capacity (IC) is essential for developing population approaches. This study examined the cross‐sectional associations of nutrition‐related, physical, and social factors with IC among community‐dwelling older adults.

**Methods:**

Participants were 822 community‐dwelling older adults. IC was assessed across five domains of locomotion, vitality, psychological capacity, cognitive function, and sensory function. Each IC domain score was log‐transformed and standardized, and a gender‐specific Confirmatory factor analysis (CFA)‐weighted composite IC score was constructed by fitting single‐factor CFA models separately for men and women. Nutrition‐related, physical, and social factors were defined according to our previous study. Participants were classified according to the quantity of lifestyle‐related factors met (0–3). Multivariate linear regression analyses were performed to investigate the association between IC score with three factors comprehensively and separately on the total sample stratified by gender.

**Results:**

The mean age was 79.0 ± 6.6 (women, 43.3%). Compared to implementing no factors, implementing two or three factors was consistently associated with significantly higher IC scores (Unstandardized Coefficient *B* [95% CI], 0.49 [0.31–0.67] and 0.68 [0.47–0.89]). Implementing one factor did not reach statistical significance after full multivariable adjustment (0.17 [−0.04–0.34]). These graded associations were broadly consistent across women and men. Moreover, factor‐specific analyses showed that nutrition‐related, physical, and social factors were all positively associated with IC in the total sample (0.22 [0.07–0.36], 0.36 [0.20–0.52] and 0.33 [0.17–0.49]).

**Conclusion:**

In this cross‐sectional study, a greater number of favorable lifestyle‐related factors was positively associated with IC among community‐dwelling older adults. These findings provide preliminary evidence to inform future longitudinal and intervention studies on healthy aging.

## Graphical abstract

This cross‐sectional study of community‐dwelling older adults found that nutrition‐related, physical, and social factors were each positively associated with intrinsic capacity. Having a greater number of favorable lifestyle‐related factors was progressively associated with higher intrinsic capacity, supporting multidimensional approaches to healthy aging.

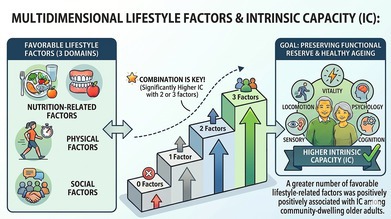

## 1 Introduction

Population aging is progressing rapidly worldwide, posing major challenges to healthcare and social security systems. In response to these challenges, the World Health Organization (WHO) introduced the concept of Intrinsic Capacity (IC) as a core component of the Integrated Care for Older People (ICOPE) framework, aiming to shift focus from disease‐centered care to the preservation of functional ability throughout the course of life [1]. IC is defined as the composite of an individual's physical and mental capacities, and comprises five key domains: locomotion, vitality, psychological capacity, cognition, and sensory function [2, 3]. Maintaining IC is fundamental for delaying functional decline, preventing dependency, and promoting healthy aging [4]. Emerging evidence indicates that lower IC is associated with adverse health outcomes, including disability, hospitalization, and mortality [2]. Therefore, identifying the modifiable factors associated with IC is a critical step toward developing effective population approaches for healthy longevity.

A growing body of literature has examined lifestyle‐related and social determinants of IC among older adults. Previous studies have reported that sociodemographic characteristics and lifestyle behaviors, including tobacco use, alcohol consumption, and physical activity, are associated with IC among older adults [5]. Physical activity and sedentary behavior have also been examined in relation to IC in population‐based studies, suggesting that higher physical activity and lower sedentary behavior may contribute to better IC or slower IC decline [6, 7]. In addition, nutrition‐related factors, including dietary patterns and healthy eating behaviors, have been associated with IC and its subdomains among community‐dwelling older adults [8, 9, 10]. Social factors, such as social frailty and social participation, have also been linked to IC in previous cohort and population‐based studies [11, 12]. More recently, studies using broader healthy lifestyle or health‐promoting lifestyle frameworks have suggested that healthier lifestyle patterns are associated with more favorable IC profiles [13].

Previous studies have reported that lifestyle factors, especially nutrition [14], oral health [15, 16], physical activity [17] and social factors [18, 19] are closely related to health outcomes among older adults. Moreover, previous studies and other epidemiological evidence have demonstrated that these three lifestyle components (nutrition‐related, physical, and social factors) are independently and synergistically associated with frailty and sarcopenia among community‐dwelling older adults [20, 21, 22]. Given that frailty and IC are related but conceptually distinct constructs, clarifying how modifiable lifestyle behaviors relate to IC is essential for advancing prevention strategies within the WHO's ICOPE framework.

However, despite the growing evidence linking lifestyle‐related factors to IC, several important gaps remain. First, many previous studies have examined individual lifestyle components, such as physical activity, diet, or social participation, separately, or have used broad composite lifestyle scores. Although these approaches are informative, they may not clarify which specific lifestyle‐related domains are independently associated with IC when considered simultaneously. Second, evidence on multidomain lifestyle approaches has mainly come from intervention studies or studies focusing on selected IC domains, whereas population‐based evidence examining daily nutrition‐related, physical, and social factors together in relation to overall IC remains limited [23]. Third, although IC and frailty are conceptually related, they are not identical constructs. Frailty often reflects accumulated vulnerability or decline, whereas IC captures the underlying physical and mental capacities that support functional ability before the onset of overt disability or dependency.

From an IC perspective, daily lifestyle behaviors are hypothesized to play a central role in maintaining functional reserves across multiple IC domains. Unlike frailty, which often manifests after substantial functional decline, IC is increasingly recognized as a key construct for understanding healthy aging within the WHO ICOPE framework. Although IC and frailty are conceptually related, IC provides a broader framework for assessing physical and mental capacities before the onset of overt disability or dependency. Therefore, identifying lifestyle‐related factors associated with IC may help generate evidence for future longitudinal and intervention studies aimed at maintaining functional ability among older adults. These behaviors may contribute to the preservation of locomotor, cognitive, psychological, and vitality domains even in the absence of overt disease or frailty. Therefore, examining nutrition‐related, physical, and social factors within the IC framework allows for a more upstream understanding of functional aging than approaches focused solely on frailty outcomes [24, 25].

This study aimed to examine the cross‐sectional associations of nutrition‐related, physical, and social factors with IC among Japanese community‐dwelling older adults using data from the Kashiwa Cohort Study. By evaluating these lifestyle‐related domains concurrently within an integrated multidomain framework, this study sought to clarify which modifiable factors are independently associated with IC and to provide preliminary evidence for future longitudinal and intervention studies aimed at maintaining functional ability and promoting healthy aging.

## 2 Methods

### 2.1 Study Design and Participants

This study employed a cross‐sectional design using data from the 2024 follow‐up survey of the Kashiwa Cohort Study, an ongoing community‐based cohort study conducted in Kashiwa City, Chiba Prefecture, Japan. The participants are residents aged 65 years or older who are living independently or receiving mild support services. Among participants in the 2024 survey (*n* = 827), individuals with MMSE scores ≤ 18 were excluded to reduce potential bias related to severe cognitive impairment and to improve the reliability of questionnaire‐based assessments [26] were excluded in the present analysis. In total, 822 participants were included in the analysis. All participants provided informed consent before participation. The study was approved by the appropriate institutional ethics committee of the University of Tokyo (approval #24‐229), and all procedures were conducted in accordance with the principles of the Declaration of Helsinki [27].

### 2.2 Measurements

#### 2.2.1 Dependent Variable: Intrinsic Capacity

Following previous studies, IC was assessed in accordance with the WHO ICOPE framework and evaluated across five domains: locomotion, vitality, psychological capacity, cognitive function, and sensory function [2].

- *Locomotion* was assessed using the Short Physical Performance Battery (SPPB), which includes balance tests (including a tandem stance), usual gait speed, and a five‐time chair stand test. Scores range from 0 to 12 points, with higher scores indicating better physical performance [28].
- *Vitality* was assessed using the Mini Nutritional Assessment (MNA), with total scores ranging from 0 to 14 [28].
- *Psychological capacity* was evaluated using the 15‐item Geriatric Depression Scale (GDS‐15), with scores ranging from 0 to 15 Higher scores indicate more severe depressive symptoms [29].
- *Cognitive function* was assessed using the Mini‐Mental State Examination (MMSE), with scores ranging from 0 to 30 [26].
- *Sensory function* was assessed using self‐reported vision and hearing. Vision was evaluated using the question, “How is your vision with both eyes at present?” (Responses of “very good” or “good” were scored as 1 point and all other responses were scored as 0 points). Hearing was assessed using the question, “Do you have difficulty hearing?” (A response of “no” was scored as 1 point and a response of “yes” as 0 points). The sensory function scores ranged from 0 to 2 points.

A composite IC score was calculated according to previous studies [30, 31]. Although the World Health Organization has proposed a multidomain framework for IC, standardized procedures for calculating a composite IC score have not yet been established. Previous studies have used various scoring approaches, including simple summation, mean domain scores, z‐score‐based indices, structural equation models, and item response theory. Therefore, in the present study, a CFA‐weighted composite IC score was constructed to reflect a single latent IC construct based on the five IC domains.

Before calculating the composite score, each IC domain score was coded so that higher values consistently indicated better capacity. Specifically, for the psychological domain assessed using the GDS‐15, the score was reverse‐coded before further processing so that a higher score represented better psychological capacity rather than more depressive symptoms. Because some domain scores included values of 0 and showed skewed distributions, all domain scores were log‐transformed after adding a constant of 1 to each value, using log (*x* + 1), where *x* represents the original domain score. This transformation allowed participants with a domain score of 0 to be retained in the analysis, since the logarithm of 0 is undefined. The log‐transformed domain scores were then standardized into z‐scores by subtracting the mean and dividing by the standard deviation of the study population, thereby allowing comparability across domains measured on different scales.

Confirmatory factor analysis (CFA) was conducted to estimate factor loadings for the five IC domains, assuming a single latent IC construct. To account for potential gender differences in the measurement structure of IC, CFA models were fitted separately for men and women. A gender‐specific weighted composite IC score was calculated as a linear combination of the standardized domain scores, using the standardized CFA factor loadings derived from the corresponding gender‐specific CFA model as weights. Specifically, the composite IC score for each participant was calculated as the sum of the products of each standardized domain score and its corresponding standardized factor loading. The five standardized domain scores represented the five IC domains, and the corresponding weights represented the relative contribution of each domain to the latent IC construct, rather than assuming equal weights across all domains.

Individual composite IC scores were calculated within each imputed dataset and then averaged across imputations to obtain the final IC score. The resulting composite IC score should be interpreted as a cross‐sectional, sample‐relative, multidimensional summary indicator of IC, with higher scores indicating better overall IC relative to the study population.

#### 2.2.2 Independent Variables: Lifestyle‐Related Factors

In this study, nutrition‐related, physical, and social factors were operationalized according to definitions used in our previous studies, which were not intended to represent clinical diagnostic criteria, but rather to identify favorable lifestyle‐related profiles that are feasible, interpretable, and potentially modifiable among community‐dwelling older adults [20]. The operational definitions were based on two main considerations: (1) the components included in each factor reflect daily lifestyle behaviors, habits, or self‐perceived functional status that are closely linked to healthy aging in community settings; (2) the criteria were designed to avoid being either too lenient or too restrictive, thereby identifying a moderate proportion of participants and improving their applicability to future community‐based population approaches.

##### 2.2.2.1 Nutrition‐Related Factors (Balanced Diet and Oral Functions)

Nutrition‐related factors comprised two components: dietary balance and oral function. Dietary balance was assessed using a food diversity score together with information on habitual protein and vegetable intake. Participants reported the frequency of consumption for 10 food groups, including meat, fish, soybean products, eggs, milk and dairy products, seaweed, green and yellow vegetables, fruits, roots and tubers, and oils and fats. Each food group was coded as 1 if consumed almost daily and 0 otherwise, yielding a total score ranging from 0 to 10 [32]. Consistent with previous research, a food diversity score of < 4 was considered indicative of low dietary diversity [33].

Additionally, frequent intake of protein‐rich foods and vegetables is crucial for older adults, especially in relation to frailty prevention [34, 35]. According to our previous study, participants were asked whether they could eat hard foods, such as squid jerky or pickled radish [20].

Based on these considerations, nutrition‐related factors were defined using three criteria: (1) a food diversity score ≥ 4, (2) almost daily consumption of meat or fish and vegetables, and (3) preserved oral function, indicated by the ability to eat hard foods. Participants were classified as meeting the nutrition‐related factor criteria if they satisfied criterion (3) and either criterion (1) or (2).

This criterion was used because preserved oral function is essential for maintaining adequate dietary intake among older adults, even when dietary diversity or intake of key food groups is favorable. Therefore, participants with preserved oral function who met either the dietary diversity criterion or the frequent intake criterion for meat/fish and vegetables were considered to have a favorable nutrition‐related profile, consistent with our previous Kashiwa cohort studies.

##### 2.2.2.2 Physical Factors

Physical factors were assessed using three items that reflected habitual physical activity and perceived physical function. Participants were asked whether they (1) engaged in exercise for at least 30 min per session, two or more times per week, for a duration exceeding 1 year; (2) walked or performed equivalent physical activities for at least 1 h per day; and (3) perceived their walking speed to be faster than that of most individuals of the same gender and age. These items were derived from the Standard Health Checkup and Counseling Guidance Program developed by the Japanese Ministry of Health, Labor, and Welfare, which includes a set of recommended indicators for evaluating population health status [36]. Participants were considered to meet the physical factor criteria if they satisfied at least two of the three criteria.

The three physical criteria reflect complementary aspects of physical behavior and perceived physical function, including regular exercise, daily walking or equivalent activity, and self‐rated walking speed. Therefore, meeting at least two criteria was considered a pragmatic threshold for identifying participants with relatively favorable physical habits and mobility‐related self‐perception, consistent with our previous Kashiwa cohort studies.

##### 2.2.2.3 Social Factors

Social factors were defined in terms of three dimensions: participation in social organizations, social support, and social networks. This stricter criterion was used because meeting only one component may reflect partial social engagement but may not sufficiently represent a favorable social environment. Therefore, the social factor was defined as the coexistence of behavioral participation, reciprocal support, and adequate network size.

Participation in social organizations was assessed by asking participants whether they participated in one or more organized activities, including older adults' clubs, sports clubs, study groups, hobbies or special‐interest groups, community, volunteer, daycare‐related activities, and other organized programs. Participation in at least one organization was considered indicative of social participation.

Social support was evaluated using the two‐way Social Support Scale, which captures both the provision and receipt of emotional and instrumental support across four items [37]. The total scores ranged from 0 to 4, with a score of 4 indicating full social support.

Social networks were measured using the Lubben Social Network Scale (LSNS‐6) [38], which assesses the size and frequency of contact with relatives and friends. Scores ranged from 0 to 30, and consistent with established thresholds, an LSNS‐6 score of 12 or higher was considered indicative of an adequate social network, whereas lower scores reflected social isolation [39].

Participants were classified as meeting the social factor criteria only if they satisfied all three components: participation in social organizations, full social support, and an adequate social network.

#### 2.2.3 Covariates

Demographic and physical characteristics, including age, gender, body mass index (BMI), and appendicular skeletal muscle mass index (ASMI), were assessed using a bioelectrical impedance analyzer (InBody 430; InBody Japan, Tokyo, Japan). Information on living arrangements was also collected, and participants were categorized as living alone or with others. The presence of chronic medical conditions, including hypertension, diabetes mellitus, osteoporosis, dyslipidemia, malignant neoplasms, and heart disease, was assessed under the supervision of a geriatrician. This information was obtained using a standardized questionnaire administered by trained nursing staff to ensure the consistency and accuracy of data collection. Moreover, gender‐stratified analyses were conducted to examine whether the associations between lifestyle‐related factors and IC scores were consistent across women and men, rather than to test or establish definitive gender‐specific effects.

### 2.3 Group Division

The participants were classified into four groups according to the total number of lifestyle‐related factors met, including nutrition‐related, physical, and social factors. The four groups were defined as follows: meeting all three factors (3 factor implemented), meeting any two of the three factors (2 factor implemented), meeting one factor (1 factor implemented), and meeting no factor (0 factor implemented). This classification was used to examine the cross‐sectional association between the number of favorable lifestyle‐related factors met and IC.

### 2.4 Statistical Analysis

Baseline characteristics of the entire sample were summarized and compared between women and men. Continuous variables were analyzed using unpaired *t*‐tests or Mann–Whitney *U* tests, depending on their distribution, whereas categorical variables were compared using chi‐square (*χ* ^2^) tests. The prevalence of nutrition‐related, physical, and social factors, as well as their individual components, was calculated for the overall sample and further stratified based on gender. Group differences were examined using chi‐square tests.

In all regression analyses, the composite IC score was treated as the dependent variable. The main independent variables were the number of favorable lifestyle‐related factors met and the individual lifestyle‐related factors, including nutrition‐related, physical, and social factors. Covariates included age, gender, living arrangements, educational level, body mass index, appendicular skeletal muscle mass index, and chronic conditions.

The assumptions of multiple linear regression were examined, including multicollinearity among the independent variables. Multicollinearity was assessed using variance inflation factors (VIFs), with VIF values below 5 considered to indicate no severe multicollinearity. To examine the cross‐sectional associations between the number of favorable lifestyle‐related factors met and IC, linear regression analyses were performed with the IC score as a continuous dependent variable, and the number of lifestyle‐related factors implemented (categorized as 0, 1, 2, or 3) as the independent variable (Table 3). As a supplementary domain‐specific analysis, we examined the associations of nutrition‐related, physical, and social factors with IC score using univariable and multivariable linear regression analyses (Table S1).

All regression models were fitted sequentially, including an unadjusted model, a model adjusted for age and gender, and a fully adjusted model further controlling for age, gender, living arrangements, educational level, and chronic conditions. Missing values in IC domain scores and covariates were handled using multiple imputation by chained equations under the missing‐at‐random assumption. The imputation model included all variables used in the analytic models. Five imputed datasets were generated, and regression estimates were pooled according to Rubin's rules. The distributions of observed and imputed values were checked to confirm the plausibility of the imputed data. All statistical analyses were performed using IBM SPSS Statistics version 28 (IBM Japan, Tokyo, Japan). Statistical significance was set at *p* < 0.05.

## 3 Results

The comparison of basic characteristics between women and men was conducted among 822 participants (mean age: 79.0 ± 6.6 years; women: 43.3%). A comparison of the characteristics of women and men is presented in Table 1. The women were slightly younger and more likely to live alone than the men. Although BMI did not differ significantly between genders, men exhibited higher appendicular skeletal muscle mass and greater grip strength, whereas women demonstrated faster usual gait speed. Regarding psychological and cognitive measures, overall cognitive function was high, and depressive symptoms were generally mild in both genders; however, significant differences in the distributions of MMSE and GDS‐15 scores were observed between women and men. Regarding chronic conditions, the prevalence of hypertension and diabetes mellitus was comparable between the genders. Osteoporosis and dyslipidemia were more prevalent among women, whereas men had a higher prevalence of heart disease and chronic renal failure. No significant gender differences were observed in the incidence of stroke or malignant neoplasms. The median (interquartile range, IQR) of the gender‐weighted intrinsic capacity score was 0.13 (−0.54 to 0.89) among men and 0.26 (−0.11 to 0.46) among women.

**TABLE 1:** Participants' characteristics stratified by gender.

	Overall		Women		Men		*p* *
No. of participants	822		356	(43.3%)	466	(56.7%)	—
Basic attributes							
Age, years	79.0	(±6.6)	78.5	(±6.6)	79.4	(±6.5)	< 0.001^a^
Living arrangements, living alone	131	(15.9%)	88	(24.7%)	43	(9.2%)	< 0.001^b^
Physical and psychological attributes							
BMI, kg/m^2^	22.8	(±3.0)	22.0	(±3.1)	23.4	(±2.9)	0.120^a^
ASMI, kg/m^2^	6.7	(±1.0)	5.9	(±0.6)	7.4	(±0.7)	< 0.001^a^
Grip strength, kg	27.7	(±7.4)	21.7	(±3.9)	32.3	(±6.1)	< 0.001^a^
Usual gait speed, m/s	1.3	(±0.2)	1.4	(±0.2)	1.3	(±0.2)	< 0.001^a^
MMSE score	29.0	(28.0–30.0)	29.0	(28.0–30.0)	29.0	(28.0–30.0)	< 0.001^c^
GDS‐15 score	3.0	(2.0–5.0)	3.0	(2.0–5.0)	3.0	(1.0–5.0)	< 0.001^c^
Present chronic conditions							
Hypertension	401	(48.8%)	169	(47.5%)	232	(49.8%)	0.603^b^
Diabetes mellitus	118	(14.4%)	43	(12.1%)	75	(16.1%)	0.116^b^
Osteoporosis	80	(9.7%)	75	(21.1%)	5	(1.1%)	< 0.001^b^
Dyslipidemia	271	(33.0%)	133	(37.4%)	138	(29.6%)	0.015^b^
Heart disease	137	(16.7%)	35	(9.8%)	102	(21.9%)	< 0.001^b^
Stroke	34	(4.1%)	15	(4.2%)	19	(4.1%)	0.899^b^
Chronic renal failure	14	(1.7%)	2	(0.6%)	12	(2.6%)	0.028^b^
Malignant neoplasm	45	(5.5%)	15	(4.2%)	30	(6.4%)	0.175^b^

Abbreviations: ASMI, appendicular skeletal muscle mass index; BMI, body mass index; GDS‐15, geriatric depression scale‐15; MMSE, mini‐mental state examination.

* *p* Values were calculated as follows: ^a^Unpaired *t*‐test;^b^ Pearson's chi‐square test; ^c^Mann–Whitney *U* test.

Across the five IC domain models, the VIF values were 1.00–1.02 for nutrition‐related factors, 1.00–1.10 for physical factors, and 1.00–1.06 for social factors. These values were well below the commonly used threshold of 5, suggesting that severe multicollinearity was not present. The distribution of nutrition‐related, physical, and social factors stratified by was examined as shown in Table 2. Regarding nutrition‐related factors, women were significantly more likely than men to report higher food diversity and more frequent consumption of meat or fish and vegetables, whereas no significant gender difference was observed in oral function, as assessed by the ability to chew hard foods. Consequently, a significantly higher proportion of women met the overall criteria for nutrition‐related factors compared with men. Regarding physical factors, men were more likely than women to report long‐term regular exercise. In contrast, no significant gender differences were observed in daily walking duration or self‐rated gait speed. Accordingly, the proportion of participants meeting the composite physical factor criteria did not differ significantly between women and men. Regarding social factors, women demonstrated significantly higher social network scores, whereas men were more likely to report full social support. Despite these differences in individual components, the overall proportion of participants meeting all social factor criteria was comparable between women and men.

**TABLE 2:** Proportions of nutrition‐related, physical, and social factors and their components, stratified by gender (*n* = 822).

Items	Total, *n* (%)	Women, *n* (%)	Men, *n* (%)	*p* *
Nutrition‐related factors (balanced diet and oral function)				
(1) Food diversity score ≥ 4	587 (71.5%)	293 (82.3%)	294 (63.1%)	< 0.001
(2) Almost daily consumption of meat or fish, and vegetables, yes	425 (51.7%)	223 (62.6%)	202 (43.3%)	< 0.001
(3) Can you eat hard foods like squid jerky or pickled radish, yes	696 (84.7%)	301 (84.6%)	395 (84.8%)	0.805
Criteria of nutrition‐related factors: (1) or (2), and (3)	366 (44.5%)	189 (53.1%)	177 (38.0%)	< 0.001
Physical factors				
(1) Exercise for 30 min or more per day, twice a week for more than 1 year, yes	500 (60.8%)	201 (56.5%)	299 (64.2%)	0.039
(2) Walking (or equivalent physical factors) for 1 h or more per day, yes	552 (67.2%)	235 (66.0%)	317 (68.0%)	0.683
(3) Gait speed is faster than for most of the people with the same gender and age, yes	472 (57.4%)	216 (60.7%)	256 (54.9%)	0.141
Criteria of physical factors: any 2 of 3 items	535 (65.1%)	226 (63.5%)	309 (66.3%)	0.515
Social factors				
(1) Participate in one or more organizational activities, yes	515 (62.7%)	235 (66.0%)	280 (60.1%)	0.054
(2) Social support score (provision and receipt), score = 4	517 (62.9%)	203 (57.0%)	314 (67.4%)	0.004
(3) Social network score, LSNS‐6 score ≥ 12	546 (66.4%)	262 (73.6%)	284 (60.9%)	< 0.001
Criteria of social factors: (1) and (2) and (3)	280 (34.1%)	126 (35.4%)	154 (33.0%)	0.421

* *p* Values were calculated using Chi‐squared tests.

The associations between the number of lifestyle‐related factors implemented (nutrition‐related, physical, and social factors) and IC scores are presented in Table 3. Compared to implementing no factors, implementing two or three factors was consistently associated with significantly higher IC scores. Implementing one factor was associated with higher IC in univariate and partially adjusted models; however, this association was attenuated and did not reach statistical significance after full multivariable adjustment. This graded association remained statistically significant after adjusting for age and gender, and persisted after further adjusting for living arrangements, educational level, and chronic conditions. Moreover, the similar results were observed by the gender‐stratified analyses, which showed broadly consistent graded associations in both women and men (Table 3).

**TABLE 3:** Association between the quantity of three factors implemented and IC score through single and multiple regression analyses.

Groups	*n* (%)	Univariate analysis		Multivariate analysis^a^		Multivariate analysis^b^	
		Unstandardized coefficient (*B*) (95% CI)	*p*	Unstandardized coefficient (*B*) (95% CI)	*p*	Unstandardized coefficient (*B*) (95% CI)	*p*
Total (*n* = 822)							
0 factor implemented	145 (17.6%)	Reference	—	Reference	—	Reference	—
1 factor implemented	294 (35.8%)	0.25 (0.08–0.43)	0.005	0.23 (0.06–0.40)	0.008	0.17 (−0.04–0.34)	0.056
2 factors implemented	264 (32.1%)	0.56 (0.38–0.73)	< 0.001	0.53 (0.36–0.70)	< 0.001	0.49 (0.31–0.67)	< 0.001
3 factors implemented	119 (14.5%)	0.76 (0.55–0.98)	< 0.001	0.72 (0.52–0.93)	< 0.001	0.68 (0.47–0.89)	< 0.001
Women (*n* = 356)							
0 factor implemented	57 (16.0%)	Reference	—	Reference	—	Reference	—
1 factor implemented	121 (34.0%)	0.26 (0.02–0.50)	0.031	0.22 (−0.13–0.45)	0.064	0.18 (−0.06–0.43)	0.145
2 factors implemented	113 (31.7%)	0.35 (0.11–0.59)	0.005	0.30 (0.06–0.53)	< 0.001	0.26 (0.02–0.50)	0.031
3 factors implemented	65 (18.3%)	0.59 (0.32–0.86)	< 0.001	0.47 (0.21–0.74)	< 0.001	0.43 (0.16–0.70)	0.004
Men (*n* = 466)							
0 factor implemented	88 (18.9%)	Reference	—	Reference	—	Reference	—
1 factor implemented	173 (37.1%)	0.24 (0.00–0.49)	0.050	0.23 (−0.06–0.47)	0.056	0.19 (−0.07–0.44)	0.131
2 factors implemented	151 (32.4%)	0.71 (0.46–0.96)	< 0.001	0.69 (0.44–0.93)	< 0.001	0.65 (0.40–0.91)	< 0.001
3 factors implemented	54 (11.6%)	0.96 (0.64–1.28)	< 0.001	0.96 (0.65–1.27)	< 0.001	0.88 (0.57–1.21)	< 0.001

Abbreviation: 95% CI, 95% confidence interval.

^a^ Adjusted by age and gender (in total).

^b^ Adjusted by age, gender (in total), living arrangements (living alone/living with others), education level (years), body mass index, appendicular skeletal muscle mass index, chronic conditions (hypertension, osteoporosis, dyslipidemia, heart disease, and stroke).

In the supplementary domain‐specific analysis, nutrition‐related, physical, and social factors were mutually adjusted for the other lifestyle‐related domains. In the total sample, all three domains remained significantly associated with IC score after full adjustment. In sex‐stratified analyses, physical and social factors remained significantly associated with IC score among women, whereas all three domains remained significantly associated with IC score among men. These results are shown in Table S1.

## 4 Discussion

In this study, we examined the associations between nutrition‐related, physical, and social factors and IC among community‐dwelling older adults, with attention to the number of favorable lifestyle‐related factors met at the time of assessment. We found that each lifestyle‐related factor was positively associated with IC, and that a greater number of favorable lifestyle‐related factors present at the time of assessment was progressively associated with higher IC scores. This graded association was consistently observed among both women and men, suggesting that the coexistence of multiple favorable lifestyle‐related factors may be linked to higher IC among community‐dwelling older adults. These findings are consistent with the view that IC is related to multidimensional lifestyle‐related profiles rather than isolated behaviors.

Consistent with prior literature, our findings support the view that healthy aging is shaped by multiple interacting lifestyle factors rather than single exposures. Integrated lifestyle approaches targeting nutrition, physical activity, and psychosocial or social components have been associated with more favorable functional and cognitive outcomes in older adults, with systematic reviews generally reporting benefits across frailty‐ and function‐related endpoints despite heterogeneity in intervention design [40]. These findings should be interpreted within the IC framework rather than as an extension of frailty research. Although frailty and IC are related, the present study focuses on IC as a dynamic and modifiable functional reserve that exists prior to clinically apparent frailty or disability. The observed associations between lifestyle‐related factors and IC suggest that everyday behaviors may contribute to the maintenance of functional capacity at an earlier stage of the aging process [25]. Observational studies have linked higher diet quality and greater dietary diversity to better cognitive performance and lower risk of cognitive impairment or decline, aligning with our results. Similarly, meta‐analytic evidence indicates that social participation and broader social relationships are associated with reduced cognitive decline or better cognitive outcomes [41, 42]. Emerging IC‐focused studies further suggest positive associations between social participation and IC‐related constructs, reinforcing the relevance of social exposures when interpreting IC as a holistic healthy aging construct [12, 43].

The lack of statistical significance for nutrition‐related factors among women shown in Table S1 may partly reflect differences in exposure distribution rather than a true gender‐specific association. Previous studies have consistently shown that women tend to engage in healthier dietary behaviors and exhibit higher overall diet quality than men [44]. In addition, women have been reported to demonstrate better oral health behaviors, including more frequent oral hygiene practices and greater utilization of dental services, particularly in older populations [45]. Consistent with these findings, women in the present study exhibited a more favorable and less variable distribution of nutrition‐related factors (Table 2), which may have reduced the statistical power to detect an independent association with IC in regression analyses. This pattern is therefore more compatible with a ceiling effect than with a lack of relevance of nutrition‐related factors among women.

From a community and public health perspective, the present findings indicate a cross‐sectional association between favorable nutrition‐related, physical, and social profiles and higher IC score among community‐dwelling older adults. However, given the cross‐sectional design of this study, these findings should not be interpreted as evidence of causal relationships, and further longitudinal data are needed to clarify the temporal direction of these associations. The observed associations may provide hypothesis‐generating evidence to inform future longitudinal and intervention studies examining whether multidimensional lifestyle‐related approaches contribute to the maintenance of IC. Although the nutrition‐related, physical, and social factors examined in this study correspond to potentially modifiable behaviors and conditions in community settings, their causal effects and practical implications for policy or intervention development should be evaluated in future prospective studies.

Several limitations should be considered when interpreting the findings of this study. First, the cross‐sectional design precludes causal inference, and reverse causation cannot be ruled out, as individuals with higher IC may be more likely to engage in healthier lifestyle behaviors. Therefore, longitudinal analyses are needed to clarify the temporal direction of these associations and to determine whether favorable lifestyle‐related profiles are associated with changes in IC over time. Second, some lifestyle‐related variables were assessed using self‐reported measures, which are subject to recall and social desirability biases and may have led to misclassification. Third, despite extensive adjustment for potential confounders, residual confounding cannot be fully excluded. Finally, the study population consisted of relatively healthy, community‐dwelling older adults from a single community, which may limit the generalizability of the findings to more frail populations or other cultural and healthcare contexts.

In this study, a greater number of favorable nutrition‐related, physical, and social factors was associated with higher IC among community‐dwelling older adults. These findings suggest that multidimensional lifestyle‐related profiles may be relevant to IC and provide preliminary evidence for future longitudinal and intervention studies aimed at maintaining functional ability and promoting healthy aging.

## Author Contributions

Weida Lyu conceived and designed the study, analyzed and interpreted the data, and drafted the manuscript. Tomoki Tanaka and Katsuya Iijima critically revised the manuscript for important intellectual content. Katsuya Iijima also oversaw project management.

## Funding

This study was supported by a Health and Labor Sciences Research Grant (Grant No.: H24‐Choju‐Ippan‐002) provided by the Ministry of Health, Labor, and Welfare of Japan; the Japan Agency for Medical Research and Development (Grant No.: JP21km0908001); the Institute for Health Economics and Policy, Japan; JSPS KAKENHI (Grant No.: 24K23744); and JSPS KAKENHI (Grant No.: 24KK0169).

## Ethics Statement

The study was approved by the appropriate institutional ethics committee of the University of Tokyo (approval #24‐229), and all procedures were conducted in accordance with the principles of the Declaration of Helsinki.

## Conflicts of Interest

The authors declare no conflicts of interest.

## Supporting information


**Table S1:** Associations of nutrition‐related, physical, and social factors with intrinsic capacity score in univariable and multivariable regression analyses.

## Data Availability

The data that support the findings of this study are available from the corresponding author upon reasonable request.
