# Supplementary material for: Cross‐Sectional Associations of Integrated Lifestyle‐Related Factors With Intrinsic Capacity in Community‐Dwelling Older Adults: The Kashiwa Cohort Study
Source: Geriatr Gerontol Int. 2026 Jul 15;26(7):e70671. doi: 10.1111/ggi.70671 (PMC13372520; doi:10.1111/ggi.70671)
Supplement: Supplementary file 1 — Table S1: Associations of nutrition‐related, physical, and social factors with intrinsic capacity score in univariable and multivariable regression analyses. [file GGI-26-0-s001.docx]

| **Supplementary Table 1. Associations of nutrition-related, physical, and social factors with intrinsic capacity score in univariable and multivariable regression analyses.** | | | | | | |
| --- | --- | --- | --- | --- | --- | --- |
| **Factors** | **Univariate analysis** | | **Multivariable model 1***^*^* | | **Multivariable model 2**^†^ | |
|  | Unstandardized Coefficient (B) (95%CI) | *p* | Unstandardized Coefficient (B) (95%CI) | *p* | Unstandardized Coefficient (B) (95%CI) | *p* |
| **Total** |  |  |  |  |  |  |
| Nutrition-related factors | 0.19 (0.04-0.34) | ***.015*** | 0.22 (0.07-0.37) | ***.004*** | 0.22 (0.07-0.36) | ***.005*** |
| Physical factors | 0.39 (0.23-0.54) | ***<.001*** | 0.36 (0.20-0.51) | ***<.001*** | 0.36 (0.20-0.52) | ***<.001*** |
| Social factors | 0.39 (0.24-0.55) | ***<.001*** | 0.32 (0.17-0.48) | ***<.001*** | 0.33 (0.17-0.49) | ***<.001*** |
|  |  |  |  |  |  |  |
| **Women** |  |  |  |  |  |  |
| Nutrition-related factors | 0.21 (-0.06-0.47) | ***.125*** | 0.23 (-0.03-0.49) | ***.079*** | 0.13 (-0.03-0.28) | ***.118*** |
| Physical factors | 0.33 (0.06-0.60) | ***.017*** | 0.25 (-0.01-0.52) | ***.061*** | 0.21 (0.04-0.37) | ***.015*** |
| Social factors | 0.35 (0.08-0.62) | ***.011*** | 0.23 (-0.03-0.50) | ***.085*** | 0.18 (0.01-0.35) | ***.034*** |
| **Men** |  |  |  |  |  |  |
| Nutrition-related factors | 0.18 (0.03-0.36) | ***.047*** | 0.22 (0.05-0.40) | ***.013*** | 0.22 (0.04-0.39) | ***.016*** |
| Physical factors | 0.44 (0.25-0.63) | ***<.001*** | 0.43 (0.25-0.61) | ***<.001*** | 0.40 (0.21-0.59) | ***<.001*** |
| Social factors | 0.41 (0.22-0.60) | ***<.001*** | 0.38 (0.20-0.56) | ***<.001*** | 0.37 (0.19-0.56) | ***<.001*** |
| *Notes: 95% CI, 95% confidence interval.*  *Multivariable model 1^*^ Adjusted for age and sex in the total sample, and mutually adjusted for nutrition-related, physical, and social factors. In sex-stratified analyses, sex was not included in the model;*  *Multivariable model 2*^†^ *Adjusted for age and sex in the total sample, mutually adjusted for nutrition-related, physical, and social factors, and further adjusted for living arrangement, education level, body mass index, appendicular skeletal muscle mass index, and chronic conditions, including hypertension, osteoporosis, dyslipidemia, heart disease, and stroke. In sex-stratified analyses, sex was not included in the model.* | | | | | | |
